# Zebrafish Thrombocytes: Functions and Origins

**DOI:** 10.1155/2012/857058

**Published:** 2012-06-24

**Authors:** Gauri Khandekar, Seongcheol Kim, Pudur Jagadeeswaran

**Affiliations:** Department of Biological Sciences, University of North Texas, Denton, TX 76203-5017, USA

## Abstract

Platelets play an important role in mammalian hemostasis. Thrombocytes of early vertebrates are functionally equivalent to mammalian platelets. A substantial amount of research has been done to study platelet function in humans as well as in animal models. However, to date only limited functional genomic studies of platelets have been performed but are low throughput and are not cost-effective. Keeping this in mind we introduced zebrafish, a vertebrate genetic model to study platelet function. We characterized zebrafish thrombocytes and established functional assays study not only their hemostatic function but to also their production. We identified a few genes which play a role in their function and production. Since we introduced the zebrafish model for the study of hemostasis and thrombosis, other groups have adapted this model to study genes that are associated with thrombocyte function and a few novel genes have also been identified. Furthermore, transgenic zebrafish with GFP-tagged thrombocytes have been developed which helped to study the production of thrombocytes and their precursors as well as their functional roles not only in hemostasis but also hematopoiesis. This paper integrates the information available on zebrafish thrombocyte function and its formation.

## 1. Introduction 

Hemostasis is a defense mechanism to prevent loss of blood in the event of an injury in an organism that has a vasculature [1]. It consists of the platelet response to injury which results in platelet aggregation and plugging the wound, termed primary hemostasis, followed by the interplay of a complex cascade of coagulation factors on the platelet surface ultimately resulting in a fibrin clot, termed secondary hemostasis. After their primary hemostatic function platelets, also repair the damaged endothelium [2]. In primary hemostasis platelets adhere to collagen in the subendothelial matrix in response to injury and are subsequently activated by a complex signaling cascade resulting in secretion of their granular contents. These contents also result in the amplification of platelet aggregation at the site of injury and formation of a platelet plug which is stabilized further with help of fibrin [1]. This hemostatic plug prevents loss of blood from the site of injury. Thus, platelets that play a role in hemostasis and defects in platelet function have been shown to be involved in bleeding disorders as well as many pathophysiological conditions like thrombosis, inflammation, and even cancer [3]. Platelets have a number of receptors on their membrane surface that help regulate signaling pathways in platelets. A substantial amount of research has been done in studying platelet development and function mostly using human platelets [2–4] murine models [4], and identification of a number of factors and their roles in platelet function [2–4]. Recently, to identify novel factors involved in platelet function, N-ethyl-N-nitrosourea (ENU) mutagenesis and genomic screens of genes affecting platelet development and function have been attempted in mice [5]. However, they are expensive, less efficient, and have lower throughput. In humans, several novel quantitative trait loci associated with platelet-signaling pathways have been identified: however, these studies require additional functional evaluation using either animal models or human subjects [6]. Thus, study of platelet function requires a model system that is efficient, less costly, and amenable to higher-throughput screen, with hemostatic pathways similar to those found in humans [7]. The hemostatic system of invertebrates differs from that of vertebrates and therefore cannot be used as a model organism to study hemostasis [8]. In this regard, we wondered whether *Danio rerio* (Zebrafish) previously used as a genetic model to study developmental biology could be used as a genetic model to study hemostasis especially platelet biology [1]. Its high fecundity, external fertilization, transparency at early stages of development, and availability of large-scale mutagenesis methods are some of the features that make it a useful model system, thus attracting our attention [9, 10]. However, the challenge was to prove whether zebrafish thrombocytes and their functional pathways are similar to those found in platelets. For this, characterization of thrombocytes and their functional pathways was required as well as technology suitable for large-scale screens. Therefore, we developed the required technologies ourselves and found them sufficient enough to warrant their utility for the study of hemostatic function. Recently, several groups utilized our zebrafish model to study hemostasis and discovered several factors regulating hemostasis [11]. This paper provides an overview on the zebrafish thrombocyte characterization and development as well as other advances made not only in our laboratory but also from other laboratories which have applied the knowledge and technology that we developed in studying thrombocyte biology.

## 2. Development of Zebrafish Model to Study Thrombocyte Function

**Figure 1:**
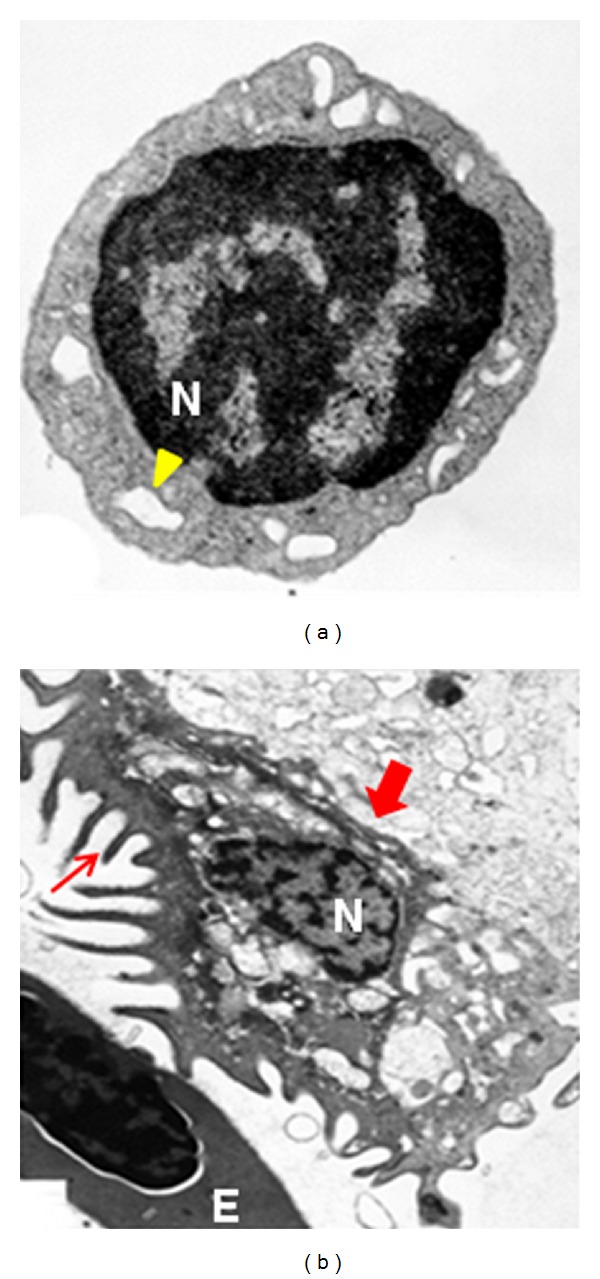
Zebrafish thrombocyte electron micrographs.  (a) Zebrafish thrombocyte. Open canalicular like system is shown by arrowhead; N: nucleus; (b) An activated thrombocyte. Thrombocyte in an aggregation reaction; activated thrombocyte is shown by a thick arrow, thrombocyte in the aggregate shows filopodia shown by a thin arrow; E: erythrocyte [12].

Unlike mammalian platelets which are anucleated, zebrafish thrombocytes have a nucleus. Our work has shown morphological and functional similarities between the zebrafish thrombocytes and human platelets [12]. Zebrafish thrombocytes have a sparse cytoplasm with large nuclei. The ultrastructure analysis of thrombocytes demonstrated that the cytoplasm contains many vesicles that open to the cell surface, similar to the open canalicular system in mammalian platelets (Figure 1). To demonstrate thrombocyte function, we developed blood collection and thrombocyte aggregation assays using less than one microliter of blood and established that zebrafish thrombocytes are stimulated by agonists including collagen, ADP, ristocetin, and arachidonic acid consistent with the human platelet aggregation methods. The results from such analyses revealed that the receptors for collagen, ADP, vWF, and thromboxane are conserved [12]. By using immunological methods, we have shown that *α*IIb integrin receptor and GpIb are present on thrombocyte membrane. Cox1 and Cox2 enzymes involved in arachidonic acid metabolism have also been identified in zebrafish [13]. Recently, we have shown that the thrombin receptor PAR-1 and its paralogue PAR-2 are also present on thrombocytes [14]. Using antibody staining and RT-PCR, we have also shown the presence of vWF in thrombocytes [15]. In a recent review, Lang et al. provide a detailed result of BLAST searches between human adhesion proteins and zebrafish proteins confirming our evidence for their similarities [16]. Thus, receptors for both thrombocyte adhesion and aggregation have been shown to be conserved in zebrafish. Subsequently, we developed a laser-induced thrombosis assay to study thrombocyte function and established that thrombosis assays are physiologically relevant in this model [17]. This study resulted in three assays, time to occlusion of artery from the time of laser injury (TTO), time to attachment of first cell from the time of laser injury (TTA) and also time taken to dissolution of the aggregate (TTD). Several reviews regarding the development of the zebrafish model for the study of thrombocyte function using laser-induced thrombosis assays from our laboratory are available [18–21]. 

## 3. Cell Biology of Thrombocyte Function

**Figure 2:**
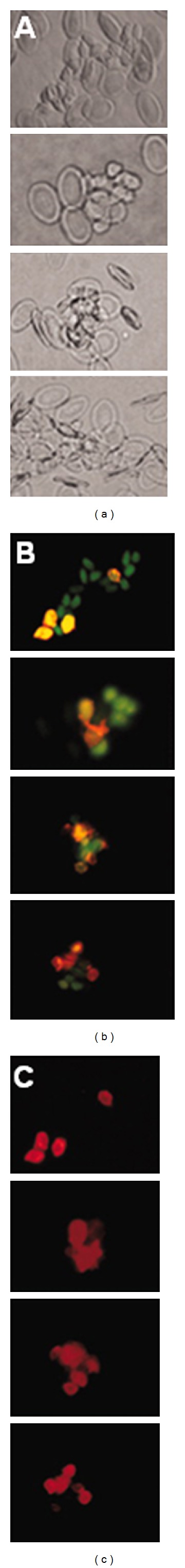
Young and mature thrombocytes forming independent clusters in an aggregation reaction. Top to bottom, the panels show four different thrombocyte clusters. (a) bright field image; (b) DiI−labeled thrombocytes and mepacrine-labeled thrombocytes as green or orange; (c) DiI-labeled thrombocytes [23].

To visualize thrombus formation, we wanted to perform intravital staining of the blood cells in zebrafish larvae by intravenous injection of lipophilic dye DiI-C18 (DiI) [22]. Surprisingly, we found only a few cells in the circulating blood were labeled in contrast to the entire blood cells. Subsequently, we identified that only a small proportion of thrombocytes in zebrafish blood was labeled by DiI alone, whereas all thrombocytes were labeled by mepacrine and, thus, giving two populations of thrombocytes (DiI+ and DiI−) (Figure 2). We found that DiI+ thrombocytes have higher levels of rough endoplasmic reticulum and thus higher protein synthesis than the DiI− thrombocytes. Furthermore, labeling the thrombocytes with BrdU for 24 hours resulted in BrdU-labeled circulating thrombocytes which were DiI+, but there were no BrdU-labeled thrombocytes that were DiI−. These results suggested that DiI+ thrombocytes were the first ones to appear in the circulation and, therefore, we called them young thrombocytes which are generated by their precursor cells by thrombopoiesis; by contrast, DiI− thrombocytes were called mature thrombocytes since in the circulation young thrombocytes presumably progress through the maturation process. By performing annexin V binding assays and estimating P-selectin levels on these two types of thrombocytes, we found that young thrombocytes are functionally more active than the mature thrombocytes [23]. In addition, we also found that young thrombocytes first appear at the site of injury and form their own clusters followed by the subsequent appearance of a mature thrombocyte cluster [23].

We have recently identified in a transgenic line initially developed by Weinstein's laboratory (National Institutes of Health, Bethesda, Maryland) for the purpose of imaging blood vessels (where GFP expression is driven by the endothelial cell-specific transcription factor, *fli1* gene promoter), circulating thrombocytes are labeled with GFP. In this line, we found two populations of thrombocytes, one DiI+, which has a less intense GFP expression, and one DiI− with a more intense GFP expression [24]. We also noted that the less intense GFP thrombocytes are first responders to injury and the more intense thrombocytes correspond to mature thrombocytes and have confirmed our previous findings using intravital microscopy [23].

## 4. Thrombocyte Microparticles

Platelet microparticles are the microvesicles released by platelets upon activation and have been shown to be involved in thrombin generation [25]. These are 0.1–1.0 *μ*m in diameter and posses most receptors found on platelets such as P-selectin, GPIb, and *α*IIb*β*3 [26]. Microparticle formation from platelets is believed to occur when the asymmetry of the membrane phospholipid is lost and phophotidylserine is externalized [27, 28]. Platelet derived microparticles are thought to promote platelet interaction with subendothelial matrix in an *α*IIb*β*3-dependent manner [29]. Elevated levels of microparticles are observed in many pathological conditions including meningococcal sepsis [30], disseminated intravascular coagulation [31], and myocardial infarction [32].

**Figure 3:**
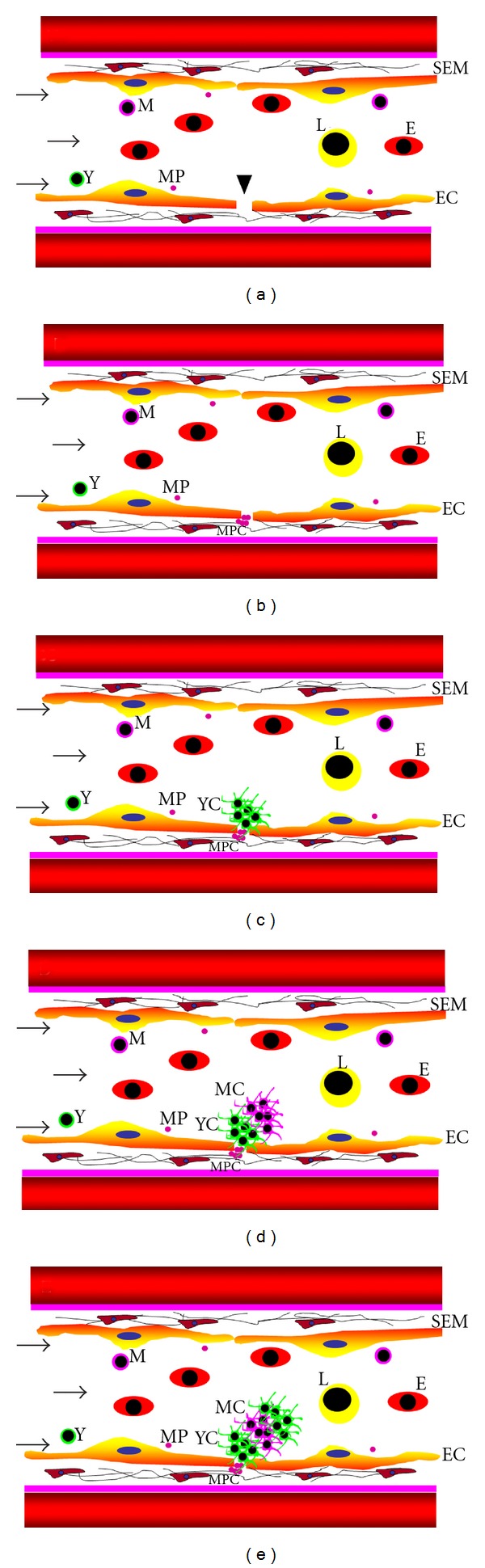
Schematic representation of sequential steps in growing arterial thrombus. Panels (a) through (e) show the sequence of events in thrombus growth. Arrowhead shows the site of laser injury in (a), (b) shows initiation of thrombus with the formation of microparticle (MP) clusters (MPC) followed by young thrombocyte (Y) clusters (YC) shown in (c) and then followed by a mixture of mature thrombocyte (M) clusters (MC) and YC as shown in (d) and (e) EC indicates endothelial cell; SE, subendothelial matrix; (e) erythrocytes; L, leukocytes. Arrows show the direction of blood flow.

We recently identified thrombocyte microparticles in zebrafish and determined that they possess the membrane protein *α*IIb, which is also found in thrombocytes. Positive labeling of zebrafish microparticles with FITC annexin V suggests that microparticles could be a result of thrombocyte apoptosis [33]. To elucidate the role of microparticles in hemostasis, Kim et al. used CD41-GFP labeled zebrafish and studied microparticle aggregation/agglutination in the presence of different agonists. Thrombin, ADP, and collagen did not aggregate thrombocyte microparticles; however, ristocetin induced agglutination in microparticles derived from thrombocytes as well as non-thrombocytes, suggesting that the agglutination is dependent on vWF. During laser injury, we have shown that the thrombocyte microparticles are the first players to arrive at the site of injury (Figure 3), even before the young thrombocytes [33]. 

## 5. Genetics and Gene Knockdowns to Study Thrombocyte Function

ENU mutagenesis has been used extensively in forward genetic screens in an unbiased manner [1]. With the laser-induced thrombosis method a relatively high throughput screen is possible to select zebrafish mutants which have hemostatic defects. We proposed that such mutagenesis methods, combined with the laser-induced thrombosis method may lead to the discovery of novel thrombocyte-specific genes and so we pursued this approach. We performed a large-scale screen and found several mutants which have hemostatic defects; however, one mutant which we characterized has a defect in a novel orphan GPCR suggesting it plays a role in thrombocyte function (manuscript in preparation). Thus, we have established it is possible to conduct forward genetic screens for hemostatic function. Another mutant which has relevance to thrombocyte function is the *fade* *out* mutant which reiterates several aspects of Hermansky-Pudlak syndrome [34]. Furthermore, in the large-scale genome TILLING project spearheaded by Sanger Institute, several mutations in genes related to thrombocyte function were found. However, these will have to be sorted out and their functional evaluation performed in the near future. 

We have also applied the knockdown technology developed by Ekker and his coworkers to study hemostatic function [35]. We used knockdown of clotting factors to establish the proof of principle and suggested that we could study the thrombocyte functions by knockdowns [1, 17, 36]. Knockdowns of thrombocyte-specific genes selected by microarray RNA analysis has resulted in identifying four genes (*acvr1, ift122, poldip2 *and* ripk5*) all of whose deficiencies, in addition to other abnormalities, gave either a hemorrhagic phenotype or prolongation of TTO phenotype [37, 38]. Since then, several knockdowns affecting thrombocyte function have appeared (see Table 1). Schulte-Merker and his group silenced myosin light chain kinase gene *mlck1a* that is expressed inthrombocytesby knockdown and found this gene is important in thrombus formation [39]. By using knockdowns and our zebrafish thrombosis model, O'Connor et al. have identified four novel genes (*bambi, lrrc32, dcbld2 *  and* esam*) involved in platelet function [11]. These genes were selected from comparative transcript analysis of platelets and megakaryocytes together with nucleated blood cells, endothelial cells and erythroblasts. In this work, they used CD41-GFP zebrafish to estimate thrombocyte aggregation during arterial thrombosis by measuring thrombocyte surface area (TSA) which essentially provides similar information as the TTO assay. Another group has also used the zebrafish model to decipher the role of *prkca *(*PKC*α**) and *prkcb *(*PKC*β**) genes in thrombocyte function; by knockdown expression of these genes, they showed that knockdown with either morpholino leads to attenuated thrombus formation [40]. This group has also used the TSA method but in their recent review they suggested that the manual TSA measurements may be time consuming and may not be accurate when using fluorescence measurements in CD41-GFP larvae although O'Connor et al. have calculated TSA for every minute of the time course and effectively used this method in their work [11, 41]. 

Although the knockdown methods combined with the laser-induced thrombosis method have the ability to demonstrate the function of the gene that plays a role in thrombosis, biochemical studies on thrombocytes cannot be performed because there is no way to study thrombocyte function by collecting blood samples from the larvae. In order to study the pathways involved in thrombocyte signaling, a knockdown in adult zebrafish was needed. Therefore, we used Vivo morpholino and created an adult Glanzmann's thrombasthenia phenotype by knockdown of the *itga2b* (*CD41*) gene [42]. With this advancement, it is now possible to study the biochemistry of thrombocytes after knockdown since we have already developed blood collection methods, thrombocyte assays and thrombocyte separation methods [14, 43, 44].

## 6. Cell Biology and Genetics of Thrombopoiesis

### 6.1. Development of Zebrafish Model for Thrombopoiesis

**Figure 4:**
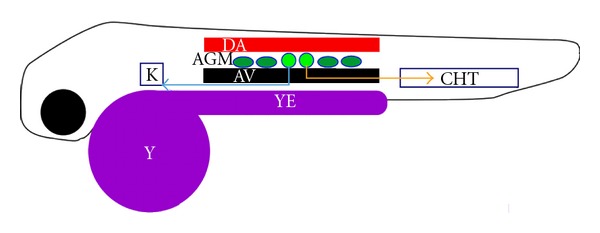
Schematic representation of thrombocyte development in zebrafish larva. DA, dorsal aorta; AV, axial vein; AGM, area corresponding to mammalian aorta- gonad- mesonephros; CHT, caudal hematopoietic tissue; K, kidney; Y, yolk; YE, yolk extension; filled small circles and ovals represent GFP^Low^ and GFP^High^ thrombocytes, respectively. The yellow and blue lines with arrows correspond to the routes of immigration of the thrombocytes. Thymus is not shown. Black circle and outline show the eye and the zebrafish body, respectively.

In zebrafish, hematopoiesis has been extensively studied [45, 46]. There are four distinct waves in the hematopoietic program of the developing zebrafish embryo. The first two waves start prior to 30 hpf in a region in zebrafish embryo called intermediate cell mass (ICM) where macrophages and erythrocytes are generated, resulting in primitive hematopoiesis. The third and fourth waves are called definitive hematopoiesis and produce erythromyeloid progenitors and hematopoietic stem cells (HSCs), respectively. The third wave may start as early as 24 hpf but peaks at around 30 hpf in caudal hematopoietic tissue (CHT) also called the posterior blood island. The fourth wave starting at 32–36 hpf occurs within endothelial cells of the ventral wall of the dorsal aorta, comparable to mammalian aorta-gonad-mesonephros (AGM). The HSCs produced from the fourth wave colonize the CHT and adult hematopoietic organs, the kidney and thymus (Figure 4). Unfortunately, at the time we began our studies with zebrafish thrombocytes zebrafish thrombopoiesis, received little attention due to the lack of labeling of zebrafish thrombocytes and the inability to follow their development. Therefore, we took advantage of labeling of circulating thrombocytes *in vivo* by intravital microscopy in order to test when thrombocytes appear in the circulation during development. We found by DiI labeling, which specifically labels thrombocytes, that thrombocytes were present in the circulation around 36 hpf, almost coinciding with the fourth wave of hematopoiesis that occurs within the ventral wall of dorsal aorta suggesting precursors for thrombocytes must exist prior to 36 hpf [22]. Subsequently, Handin's laboratory developed a transgenic zebrafish (CD41-GFP zebrafish) where they used the *CD41* gene promoter to drive GFP expression. In this line they found green fluorescent cells flowing in the blood stream around 48 hpf; after this observation they asked us to test whether these cells aggregate using our thrombosis and thrombocyte aggregation assays. When we performed aggregation assays and laser injury thrombosis assays, a green fluorescent aggregate formed, establishing that Handin's green fluorescent cells were in fact thrombocytes [47]. Furthermore, it also provided the possibility of quantifying the intensity of the thrombocyte aggregates. Subsequently, knockdown of transcription factor gene *scl* and the receptor for a cytokine thrombopoietin *c-mpl* gene resulted in reduction of GFP-labeled thrombocytes, suggesting the presence of C-mpl receptor on zebrafish thrombocytes [48]. C-mpl receptor mRNA was shown to be present in the thrombocytes as early as 42 hpf [49]. Using this transgenic line, Lin et al. determined that the GFP+ thrombocytes were not present in the ICM and, therefore, are not part of primitive hematopoiesis [48]. However, they found nonmobile GFP+ thrombocytes between the dorsal aorta and caudal vein at 40 and 48 hpf that they suggested to correspond to the AGM although not having classical AGM features. The circulatory GFP+ thrombocytes appeared first at 48 hpf [48]. FACS analysis of the GFP+ cells from mesonephros detected two distinct populations: one with bright fluorescence (GFP^High^), considered to be well-differentiated with typical thrombocyte morphology (scant cytoplasm and spindle shape), and the other with weak fluorescence (GFP^Low^) and larger than GFP^High^ thrombocytes with undifferentiated morphology (round) and basophilic cytoplasm. Further studies by Kissa and coworkers revealed that the GFP^Low^ cells appeared first at 33 to 35 hpf as single cells between the dorsal aorta and the postcardinal vein and they migrate subsequently to CHT and thymus via the axial vein rather than dorsal aorta [50]. Bertrand and his colleagues refined these studies and found that these cells appear as early as 27 hpf in the trunk randomly between axial vessels and confirmed their migration to CHT, thymus and pronephros along with the finding of their migration along the pronephric tubules [49]. These immigrants to kidney supposedly initiate adult hematopoiesis in the developing kidney. They also found migration of the GFP^Low^ cells between axial vessels and pronephric ducts and back to vessels. A recent study from Handin's laboratory revealed that the GFP^Low^ cells injected into irradiated adult zebrafish showed production of GFP+ cells in kidneys by long term multilineage reconstitution, suggesting that they have the features of HSCs while GFP^High^ cells did not reconstitute [51]. 

### 6.2. Identification of Factors Affecting Thrombocyte Development

Several transcription factors such as Fli-1, Fog1, GATA-1 (Zg1), NFE2, and Runx1 which have been found in megakaryocytes have also been identified in zebrafish [46]. *runx1* morpholino injected zebrafish embryos lack a normal circulation and accumulate immature hematopoietic progenitors [52]. The CD41-GFP cells were also found to express Runx1. Using CD41-GFP zebrafish, the truncated Runx1 developed normal CD41+ HSCs, indicating there is a Runx1-independent secondary pathway to generate HSCs [53]. Another factor, c-Myb a negative regular of megakaryocytopoiesis, has been identified in zebrafish [54]. Functional knockdown of *miR-126*, a key regulator of *c-myb* in zebrafish, resulted in an increase in erythrocytes and a decrease in thrombocytes, proving that the cell fate decision is regulated by the micro RNA [55]. Yet another factor Fog1, a cofactor that interacts with GATA-1 and GATA-2 has been shown to play a role in erythroid and megakaryocyte differentiation [56]. *fog1* morpholino injected in CD41-GFP zebrafish embryos failed to generate GFP+ mature thrombocytes suggesting that Fog1 is necessary for thrombocyte development [57]. Recently, thrombocyte maturation in the circulation has been studied in adult zebrafish, revealing that the *gata1* promoter becomes weaker and *fli1* promoter gets stronger in mature thrombocytes and is conversely regulated in young thrombocytes [24]. 

In addition to these studies, a transient knockdown of *mastl* inzebrafishresulted in deficiency of circulating thrombocytes [58]. More recently, knockdowns of genes, *march2*, *max*, *smox*, *pttg1lp*, *emilin1*, and *sufu* resulted in a severe decrease in the number of thrombocytes indicating that these genes are important for thrombocyte development [59]. These genes were selected for knockdowns by genomewide analysis studies (GWAS) for genes adjacent to binding sites for GATA-1, GATA-2, Runx1, Fli-1, and SCL using primary human cells. Another study by Gieger et al. used meta-analyses of GWAS for mean platelet volume and platelet count and identified 68 genomic loci and from these loci four genes (*arhgef3*, *ak3*, *rnf145,* and *jmjd1c*) were silenced in zebrafish which led to the ablation of both primitive erythropoiesis and thrombocyte formation. Silencing of *tpma*, the orthologue of *tpm1* transcribed in megakaryocytes but not in other blood cells, abolished the formation of thrombocytes, but not erythrocytes [60]. In addition to these findings, silencing of *nbeal2* and *rgs18* inzebrafish resulted in reduction in thrombocyte formation [61, 62]. The silencing of genes by knockdown methods affecting thrombocyte formation is summarized in Table 1. 

## 7. Future Studies

Despite the advances in genetic studies of thrombocyte function and development in zebrafish, many novel genes involved in thrombocyte origins and functions remain to be identified. For example, even though embryonic GFP^Low^ thrombocytes have been identified as HSCs and their role in repopulating the kidney for initiating the subsequent generation of thrombocytes from HSCs has not yet been investigated. Thus, we have no information regarding genes involved in the production of thrombocyte precursor cells in adult zebrafish. Likewise, studies of genes involved in maturation from young to mature thrombocytes, as well as genes controlling the production of thrombocyte microparticles are in the beginning stages. Since our laser-induced thrombosis assays for studying hemostasis have already found applications, we anticipate more such studies of this kind will be performed to assess the role of novel human genes relevant to hemostasis and thrombocyte development and function [35]. Recently developed technologies such as Genome TILLING [63, 64], zinc finger nuclease, or other nuclease/s (TALEN) based knockout methods [65–68] are also anticipated to complement the already available methods for studying functions of genes involved in thrombocyte function and production. However, large-scale silencing of genes to study thrombocyte development and production are still prohibitively expensive. Thus, future development of cost-effective gene silencing methodologies is required to attempt a functional genomics approach to analyze thrombocytes using the zebrafish model. Once the genes are identified, utilizing Vivo morpholino technology, we predict that characterization of the phenotypes by thrombocyte aggregation/adhesion functional assays, and determination of their mechanism of action will all be within reach in the next decade.

## Figures and Tables

**Table 1:** Summary of the silencing of genes by knockdown methods affecting thrombocyte formation.

Gene	Functional evaluation	Phenotype	Reference
*acvr1*	Laser thrombosis	Hemorrhagic/Prolonged TTO	[36, 37]
*ift122*	Laser thrombosis	Hemorrhagic/Prolonged TTO	[36, 37]
*poldip2*	Laser thrombosis	Hemorrhagic/Prolonged TTO	[36, 37]
*ripk5*	Laser thrombosis	Hemorrhagic/Prolonged TTO	[36, 37]
*mlck1a*	Laser thrombosis	Prolonged TTO	[39]
*bambi*	Laser thrombosis	Prolonged TTA/reduced thrombus surface area	[11]
*lrrc32*	Laser thrombosis	Prolonged TTA/Reduced TSA	[11]
*dcbld2*	Laser thrombosis	Increased TSA	[11]
*esam*	Laser thrombosis	Increased thrombus size	[11]
*prkca (PKCα*)	Laser thrombosis	Reduced TSA	[40]
*prkcb (PKCβ*)	Laser thrombosis	Reduced TSA	[40]
*itga2b (CD41)*	Laser thrombosis/thrombocyte aggregation assays	Reduced TSA/Prolonged TTO/no aggregation of thrombocytes	[11, 41]
*scl *	Thrombocyte formation	Reduction in GFP+ cells in CD41-GFP transgenic zebrafish line	[48]
*c-mpl*	Thrombocyte formation	Reduction in GFP+ cells in CD41-GFP transgenic zebrafish line	[48]
*runx1*	Whole mount in situ hybridization/immunostaining	Accumulation of hematopoietic progenitors	[52]
*miR-126/c-myb*	Thrombocyte formation	Decrease in CD41 : EGFP+ thrombocytes in a double transgenic reporter line Tg (cd41 : EGFP) : Tg (gata1 : dsRed)	[55]
*fog1*	Thrombocyte formation	Failure to generate eGFP+ cells in CD41-GFP transgenic zebrafish line	[56]
*mastl*	Thrombocyte formation	Reduction in GFP+ cells in CD41-GFP transgenic zebrafish line	[58]
*march2*	Thrombocyte formation	Reduction in GFP+ cells in CD41-GFP transgenic zebrafish line	[59]
*max*	Thrombocyte formation	Reduction in GFP+ cells in CD41-GFP transgenic zebrafish line	[59]
*smox*	Thrombocyte formation	Reduction in GFP+ cells in CD41-GFP transgenic zebrafish line	[59]
*pttg11p*	Thrombocyte formation	Reduction in GFP+ cells in CD41-GFP transgenic zebrafish line	[59]
*emilin1*	Thrombocyte formation	Reduction in GFP+ cells in CD41-GFP transgenic zebrafish line	[59]
*sufu*	Thrombocyte formation	Reduction in GFP+ cells in CD41-GFP transgenic zebrafish line	[59]
*arhgef3*	Thrombocyte formation	Absence of GFP+ cells in CD41-GFP transgenic zebrafish line	[60]
*ak3*	Thrombocyte formation	Absence of GFP+ cells in CD41-GFP transgenic zebrafish line	[60]
*rnf45*	Thrombocyte formation	Absence of GFP+ cells in CD41-GFP transgenic zebrafish line	[60]
*jmjd1c*	Thrombocyte formation	Absence of GFP+ cells in CD41-GFP transgenic zebrafish line	[60]
*tpma*	Thrombocyte formation	Absence of GFP+ cells in CD41-GFP transgenic zebrafish line	[60]
*nbeal2*	Thrombocyte formation	Abrogation of thrombocyte formation	[61]
*rgs18*	Thrombocyte formation	Thrombocytopenia	[62]
